# Determination and Calculations of Mercury Vapor Concentration and Energy Released from Freshly Condensed Dental Amalgams Having Various Copper Percentages within the Alloy

**DOI:** 10.3390/ma16093452

**Published:** 2023-04-28

**Authors:** Ryan Moxon, Zhigang Xu, Ikenna Chris-Okoro, Sheilah Cherono, Dhananjay Kumar

**Affiliations:** Department of Mechanical Engineering, North Carolina A&T State University, Greensboro, NC 27411, USAicchrisokoro@aggies.ncat.edu (I.C.-O.); scherono@aggies.ncat.edu (S.C.)

**Keywords:** mercury vapor concentration, γ_1_-phase, γ_2_-phase, phase formation, grain boundary structure, properties, ductility, hardness, corrosion resistance, creep, mercury emission

## Abstract

Dental amalgam is an alloy consisting of a mixture of fine metallic powder of silver, tin, zinc, copper, and a trace amount of palladium in combination with about fifty percent elemental mercury that forms a matrix phase. Dental amalgams consisting of a high-copper content are the most common types of alloys currently utilized for the restoration of decayed, broken, and fractured posterior human teeth. The present research objective was primarily to improve the material properties by determining and analyzing the amount of mercury vapor released from dental amalgam received from eight different commercial brands. The mechanical hardness of the alloys was found to increase with an increase in copper content in the amalgam. The effect of copper addition on material aging was also studied. During the release of mercury vapor, the corresponding energies associated with the release of mercury vapor from each sample were determined for each successive measurement. The results indicated that increasing the copper content of the amalgam counters the release of mercury vapor from posterior teeth and improves the hardness properties.

## 1. Introduction

Dental amalgam material has been utilized by dentists for the restoration of mainly posterior teeth in humans since about 1833, when it was introduced in the United States from England [1]. There are other dental restorative materials than amalgams being used today, but dentists choose to use dental amalgam for posterior teeth restoration due to its higher relative strength and cost-effectiveness compared to composite resin [1,2]. In 2008, the use of dental amalgam was banned in countries such as Norway, Sweden, and Denmark due to concerns regarding mercury vapor toxicity [3]. In the United States, the American Dental Association (ADA) and the Government approved amalgam’s use in dentistry, as major manufacturers are motivated by sheer profit and dental amalgam’s affordability to customers [4]. Dental restorative material has been known to release mercury vapor at very high levels of concentration when triturated and during subsequent condensation as part of cavity preparation in human teeth [3]. The silver in the alloy is responsible for the setting expansion and creates good atomic bonding with tin, enabling an increase in strength and resistance to corrosion within the alloy [4]. More fundamentally, the silver renders the material adapted to high surface shine when polished after placement in the tooth-prepared cavity by the dentist. The tin usually causes setting contraction and gives the alloy its malleability property. Copper improves strength, minimizes corrosion and tarnishing, reduces creep, and improves marginal leakages. Zinc is the scavenger element that reduces oxidation and enables the removal of impurities in the alloy [5,6].

The classification of dental amalgam falls into two categories: (1) low-copper amalgam (about 6% Cu, conventional alloy), and (2) high-copper amalgam (>6% Cu) [6]. Low-copper amalgam has a composition of 55–60 wt. % silver, 20–29 wt. % tin, 6–8 wt. % copper, and <2 wt. % zinc [6]. The chemical formula of the amalgam is given as Ag_3_Sn (silver–tin γ) + Hg (elemental-mercury) → Ag_3_Sn (silver–tin γ phase) + Ag_2_Hg_3_ (silver–mercury γ_1_ phase) + Sn_8_Hg (tin–mercury γ_2_ phase) [6]. This takes place with a reduction in the size of the particle as the solubility of Sn and Ag in Hg is limited (0.35 and 0.6 wt. %, respectively) [4,7,8,9]. The silver precipitates out initially as Ag-Hg (γ_1_ phase), followed by tin in the form of the Sn-Hg (γ_2_ phase). The set amalgam consists of core γ particles surrounded by a matrix of γ_1_ and γ_2_ phases [10,11].

In low-copper alloys, the anodic potential of the Sn_8_Hg (γ_2_ phase) is greater than that of the Ag_2_Hg_3_ (γ_1_ phase), and this leads to corrosion and resultant distribution of the γ_2_ phase; thus, forming the reaction products that develop a protective layer of oxide and hydroxide on the outer surface of the amalgam due to aging [12,13]. It has been established conclusively that this founding phenomenon helps in the deceleration of the rate of oxidation. Mercury in the Sn_8_Hg (γ_2_ phase) is released either as mercury vapor or as solutes in the saliva. Since the Sn_8_Hg (γ_2_ phase) occupies about 15% vol. of the mixed alloy as a continuous or near-continuous structural component, the corrosion loss of this component weakens the matrix and renders it more susceptible to either marginal breakdown or possible failure, including brittleness. Porosity, which usually exists to some extent in most amalgams, accelerates the corrosion of the material [14].

High-copper amalgam has a composition of 40–60 wt. % silver, 12–30 wt. % tin, 8.5–33 wt. % copper, 0.5–9 wt. % indium, and 1–3.5 wt. % zinc, and in some amalgam alloys, it has a trace amount of palladium (0.7 wt. %) [15]. Researchers have suggested that high-copper amalgam must have a minimum of 12 wt. % copper for the elimination of the γ_2_ phase, which causes an emission of a high concentration of mercury vapor from the alloy [16,17,18,19]. The development of high-copper amalgam by adding silver–copper eutectic particles to traditional silver–tin lathe-cut particles while dispersion hardening the alloy is reported to produce improved physical properties [20]. However, these are not the result of the dispersion hardening, as the silver–copper eutectic particles were big and too spaced out to inhibit dislocation movement. Instead, these improved properties were due to the formation of Cu_6_Sn_5_ η-phase [20,21,22]. The great affinity of tin for copper ensures that the γ_2_-phase is significantly reduced or partially eliminated in addition to generating visible improvements in physical properties such as increased strength, less tarnishing, corrosion resistance, and creep reduction.

Increased levels of weight percent copper present in dental amalgam alloy [23] enable the interdiffusion of copper into the elemental mercury upon trituration, thus initiating a reaction of Sn_8_Hg (γ_2_-phase). This γ_2_-phase is eliminated through the addition of copper to the alloy. Such methods of transformation occurred through changes in the Cu_3_Sn (ε-phase) [24,25]. The concept of enthalpy and the Gibbs free energy of the reaction and phase product are considered and utilized for the calculation of the energies released at each time interval during mercury vapor measurements. The two main phases that precipitate subsequent to trituration are Ag_2_Hg_3_ (γ_1_-phase) and Sn_8_Hg (γ_2_-phase) [24]. Such precipitates initially coexist with the liquid mercury for a short period of time, approximately 10–15 min, and the mixture maintains a plastic consistency, which allows for the placement and shaping of the amalgam during its condensation into the tooth-prepared cavity. The metallic powder is made to be mixed in correct proportion with liquid mercury (consisting of 40–50 wt. %). During trituration, the mercury becomes supersaturated with the metallic atoms, thus leading to nucleation and the growth of the distinct phases which eventually precipitate from the alloy solution [26,27,28]. 

The alloy particles are manufactured in micro-cut, fine-cut, and coarse-cut particle sizes. The alloy generally develops phases in the form of binary phases, Ag-Sn (γ-phase), Ag-Sn-Cu (ternary phase), and Ag-tin-Cu-Zn (quaternary phase) [29]. The solubility of copper in the Ag_3_Sn (γ-phase) is only 1 wt. %. Therefore, excess copper forms the copper-rich phase. The amount and type of phase may vary due to the thermal processing, including the Cu_3_Sn (ε-phase) and the Cu_6_Sn_5_ (ζ-phase). High-copper admixed alloy contains a eutectic microstructure of an Ag-rich γ-phase and a copper-rich γ-phase [30]. Zinc is present in the low-copper amalgam powder, and its concentration exceeds the solubility limits of 1.6 wt. % Ag-Sn (β-phase), and 5.9% subsequently forms Cu_5_Zn_8_ [31,32].

During the amalgam aging process, changes in the composition and microstructural phases occur. The reaction of mercury and mercury-rich amalgam continues during the setting. High-copper amalgam produces a lesser amount of γ_2_-phase and transforms into the Cu_6_Sn_5_ phase. Amalgams that have been in place for many years usually present some transformation of the Ag_2_Hg_3_ (γ_1_-phase), subsequently resulting in the Ag_9_Hg_11_ (β_1_-phase) [33]. This loss can occur due to three possible mechanisms:

1. Dissolution of mercury at the amalgam surface.
2. Evaporation from the exposed surface of the amalgam.
3. Migration of mercury to the interface of the remaining portion of the unreacted high-copper particles [34].

Two other phase changes generally seem to manifest and are likely to occur during the aging process besides those associated with corrosion, one of which is the reaction of the deleterious Sn_8_Hg (γ_2_-phase) phase with unreacted Cu_3_Sn to form the Cu_6_Sn eta-phase [24]. Such will occur in the copper alloy if there is any Sn_8_Hg (γ_2_-phase). The other likely change is that of the Ag_2_Hg_3_ (γ_1_-phase) into Ag_9_Hg_11_ (β_1_-phase) [24]. This phenomenon has been observed in high-copper alloy surfaces where corrosion occurred. Diffusion of mercury frequently occurred during transformation of the alloy at room temperature during setting process. Diffusion of mercury vapor originates from the Ag_2_Hg_3_ γ_1_-rich mercury phase, which regularly transforms into the Ag_9_Hg_11_ β_1_ phase [34,35,36,37]. 

This study examines the microstructural changes of the material with advanced aging. Previous research has shown that when the material slowly undergoes oxidation, the mercury vapor levels decrease as a function of time and the oxidative process [37,38]. Such factors control the material properties, such as compressive strength, ductility, hardness, corrosion resistance, creep, and mercury vapor emission [39]. The research objective was primarily to determine and analyze the amount of mercury vapor released from each brand of dental amalgam, having different copper contents, with equal time duration of vapor measurement.

## 2. Materials and Equipment

Amalgam brands of capsules in spells of 400 mg, 600 mg, and 800 mg were obtained from commercial distribution dental supply companies such as Henry-Schein Dental (Melville, NY, USA; www.henryschein.com), Darby Dental Supply (Jericho, NY, USA; www.darbydental.com), and Patterson Dental Supply. The amalgam capsules obtained from each of the three suppliers are as follows: (1) Dispersalloy (11.8% Cu, regular-set, 2-spills, 600 mg, blue/white color capsule), (2) Permite C/SDI (15.4% Cu, regular set, 2-spills, 600 mg, purple/gray color capsule), (3) Valliant PHD (20% Cu, regular set, 2-spills, 600 mg, blue color capsule), (4) Megalloy EZ (25% Cu, 56% silver, regular set, 2-spills, 600 mg, purple color capsule), (5) Tytin FC/Kerr (26% Cu, regular set, 2-spills, 600 mg, blue/white color capsule), (6) Tytin/Kerr (28% Cu, regular set, 2-spills, 600 mg, white/yellow color capsule), Tytin/Kerr (28% Cu), (7) Contour/Kerr (31% Cu, regular-set, 2-spills, 600 mg, brown/gray color capsule), (8) Sybralloy/Kerr (33% Cu. regular-set, 2-spills, 600 mg, green/gray colored capsule).

In order to measure the concentration of mercury vapor from freshly condensed dental amalgams of various brands and the energy released at each stage of measurement, a Jerome J505 Mercury Vapor Analyzer (Arizona Instrument, Chandler, AZ, USA) was used during the experimental procedure. The Jerome J505 Instrument works on the principle of drawing an inflow of saturated air with particulates via a built-in pump. The air is made to flow over a gold-metallic strip at a warm temperature. Since gold has a high affinity for mercury atoms, the mercury atoms in the saturated air enter the instrument through an orifice by way of a 12-inch plastic tubing attached to the instrument [40].

The standard unit range for the Jerome J505 Mercury Vapor Analyzer is ng/m^3^ (50 to 500,000), μg/m^3^ (0.05 to 500), and mg/m^3^ (0.00005 to 0.50000). The instrument automatically computes statistical parameters such as the variance, standard deviations, and percentage error in the data obtained. Percentage error indicated ±0.05%, each successive measurement demonstrated similarities in results, and each measurement was obtained under normal conditions [40]. In order to understand the phase purity and orientation of the Dispersalloy and Sybralloy, X-ray diffraction analysis was performed using the Bruker D8 (Billerica, MA, USA). 

## 3. Methodology

The research investigated various samples of dental amalgam prepared first by trituration followed by condensation into samples with diameters of 10 mm andthicnkesses of 4 mm. For this research, eight of the most common brands of dental amalgams (having varying powder compositions) were obtained from commercial manufacturers: Dispersalloy—11.8% Cu, Permite C/SDI—15.4% Cu, Valliant PHD—20% Cu, Megalloy EZ—25% Cu, Tytin FC/Kerr—26% Cu, Tytin/Kerr—28% Cu, Contour/Kerr—31% Cu, Sybralloy/Kerr—33% Cu. 

Each sample was polished using rough, medium, and smooth-grade Emory polishing paper and was measured under similar environmental conditions and operating speed (temperature between 20–30 °C at 1 atm). These amalgams were distributed in capsules, and inside this capsule was a thin plastic/polymer membrane that separates the metallic powder on one side from the liquid mercury on the other side. Prior to trituration, the amalgam capsule was inserted between the two vibrating prongs of the Zenith dental variable speed amalgamator, which features a torque motor, having the choice of three speed settings: high (4800 rpm), medium (4200 rpm), and low (3600 rpm). The amalgamator was connected to a 120-vold power supply. At the onset of electric power, the amalgam capsule was allowed to vibrate at this high speed for 15 s for the trituration process of mixing. 

Prior to initiating measurement, the Mercury Vapor Analyzer was warmed-up for about 10 min, and a test sample reading was obtained in order to verify the accuracy of the measurements. Amalgam capsules were then condensed and prepared in uniform size. The samples were inserted into a Stony Lab 250 mL borosilicate glass (Nesconset, NY, USA). A 12-inch length of plastic tubing with a diameter of 4 mm was attached to the Stony Lab glass, while the other end was attached to the J505 instrument. At the onset of operations, saturated ambient air containing mercury atoms was drawn through a 12-inch plastic tube with a diameter of 4 mm attached to the 250 mL flask. Saturated air samples containing mercury atoms were drawn into the instrument by means of a built-in electrical pump located inside the instrument. The normal flow rate of saturated air is 1 L/min. The sample air then flows through a scrubber filter and directly into the sample cell located inside the instrument; the entrance of the sample cell is controlled by a one-way valve to prevent back-flow.

Six weeks subsequent to sample preparation, the hardness of each sample was measured using the Microvickers Hardness Tester, Model M-400-H1 (Mitutoyo, Kawasaki, Japan). 

Thermodynamic calculation methods were applied in determining the energies released from each sample’s stage of concentration measurement (see Equation (1) [29]. All of the measurements were obtained at room temperature during the research experiment (temperature between 20–30 °C at 1 atm). 

The study also examined the microstructural changes of the material with advanced aging by analyzing the X-ray diffraction pattern obtained from both the Dispersalloy and the Sybralloy using the D8 Bruker. 

## 4. Results and Discussions

**Figure 1:**
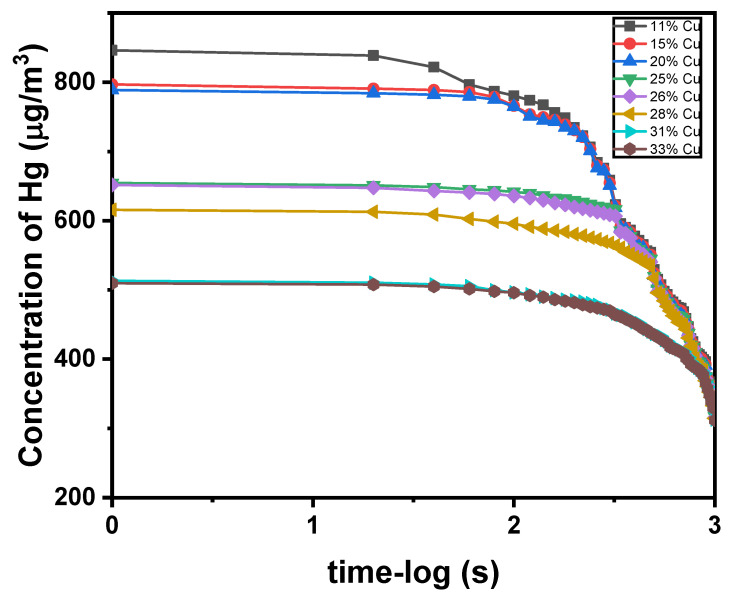
Decrease in the mercury vapor concentrations of dental amalgam samples as a function of time for the first 1000 s.

Table 1 below shows the results recorded from the Mercury vapor analyzer. In accordance with the results, and as demonstrated by Figure 1 below, the amalgam samples released a significant amount of mercury vapor. The Dispersalloy (11.8% Cu) released the highest concentration of mercury vapor, while Sybralloy (33% Cu) released the lowest amount of vapor. The vapor release measurement was conducted starting at zero seconds followed by an interval of 20 s up to 2800 s. 

### 4.1. Mercury Vapour Concentration

The results from Table 1 show the decrease in mercury vapor released with the passage of time and with the increase in weight percent copper in each sample.

**Figure 2:**
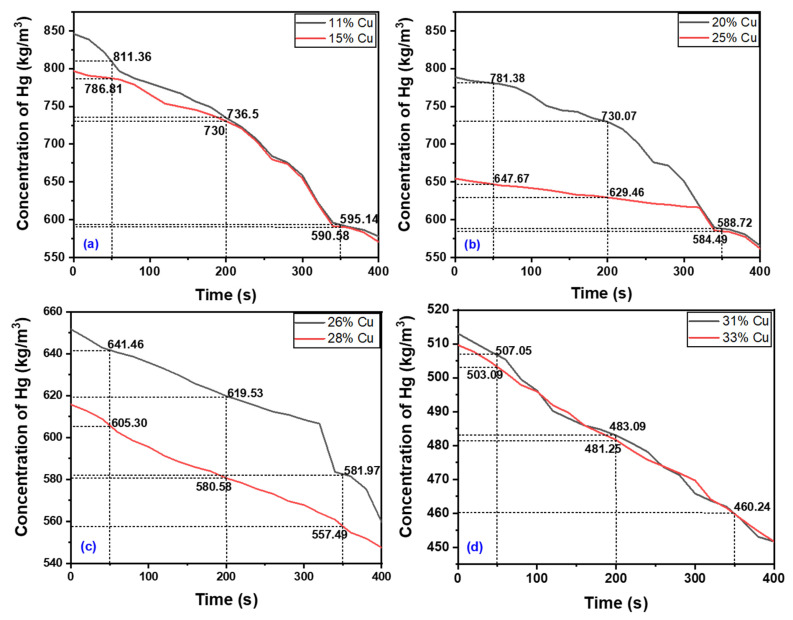
Comparison of the amount of mercury vapor released from eight amalgam samples with different Cu contents (indicated in the figures) as a function of time: (**a**) Disperse and Permite, (**b**) Valiant PHD and Megalloy EZ, (**c**) Tytin FC/Kerr and Tytin/Kerd, and (**d**) Controur and Sybralloy dental amalgam.

At the starting time (zero seconds) of the mercury vapor measurement of each alloy, the mercury vapor level for the Dispersalloy (11.8% Cu) gave a value of 846 kg/m^3^ compared to the concentration value of 796 kg/m^3^ for the Permite C/SDI (15.4% Cu) brand. For the same starting time for the Permite C/SDI (15.4% copper), the mercury released was much lower in value than that of the Dispersalloy amalgam. This is a verification that the amalgam alloy with the lowest percentage of copper will release the greatest concentration of mercury vapor, because the increased amount of copper added to the alloy will suppress the release of mercury vapor. Such evidence of the mercury vapor released from each amalgam alloy can be seen in the plots of Figure 1 and Figure 2, showing the mercury released for each alloy at intervals of 50 s, 200 s, and 350 s.

As observed from the plot of Figure 2, both the mercury vapor levels released from the Contour (31% Cu) amalgam and the Sybralloy (33%) amalgam appear to be about the same. Both alloys have approximately the same amount of copper content in their compositions, thus, each alloy releases that amount of mercury vapor in very close proximity. For the high-copper amalgam alloys such as Contour and Sybralloy, the much-increased copper content serves to eliminate the γ_2_ phase which is responsible for inducing corrosion and tarnishing within the alloy [41,42]. 

### 4.2. X-ray Diffraction Analysis

The X-ray diffraction pattern of Dispersalloy (11.8% Cu) and Sybralloy (33% Cu) shows the relative comparison of the Sn8Hg (γ_2_-phase), of which a greater amount is present in the Dispersalloy plot, as is demonstrated in Figure 1. The major phase in each the Dispersalloy (11.8% Cu) and Sybralloy (33% Cu) brands is the silver–tin (γ-phase), the strongest phase having the tallest peak. The mercury vapor is released during the γ_1_-phase (Ag_2_Hg_3_) and mostly from Sn_8_Hg (γ_2_-phase), which is predominant in the low-copper alloy. The Sybralloy (33% Cu) from Figure 1 showed a reduction in the γ_2_-phase due to the higher weight percent copper in that alloy. Interestingly, 15% vol. of the matrix phase is composed of Sn_8_Hg (γ_2_ phase), which is usually a stable phase [32]. This means that the lesser amount of copper enables strong bonding and is brooched in the Cu_3_Sn ε-phase or Cu_6_Sn_5_ η-prime phase [32,33].

**Figure 3:**
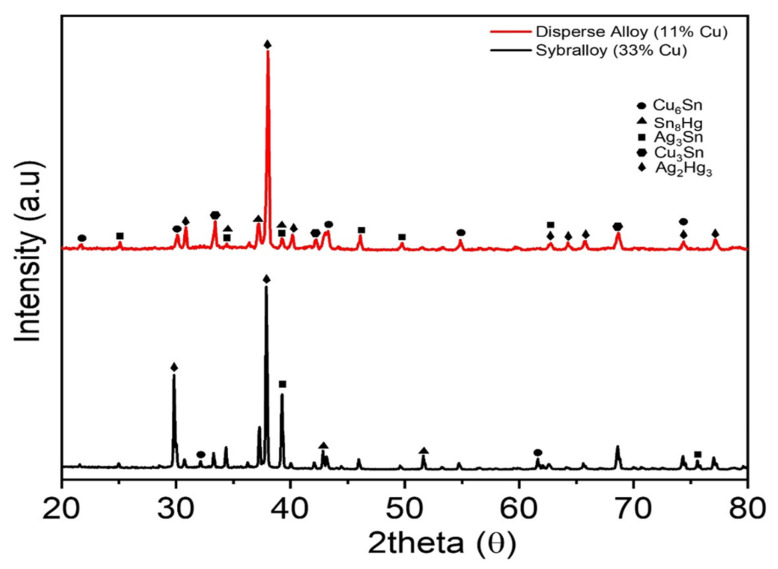
XRD pattern for the Dispersalloy (11.8% Cu) and Sybralloy (33% Cu) dental amalgams.

The X-ray diffraction image for Dispersalloy as shown in Figure 3 shows a large accumulation of the γ_2_-phase, and the X-ray diffraction for Sybralloy showed a lesser amount of the γ_2_- phase, thus indicating a lesser amount of mercury vapor released from Sybralloy than from Dispersalloy and for the other amalgam samples. Previous research has shown that the material slowly undergoes oxidation, and the mercury vapor levels decrease as a function of time and the oxidative process [37]. Such factors control the material properties, such as compressive strength, ductility, hardness, corrosion resistance, creep, and mercury vapor emission [39].

Attempts have been made to reduce the γ_2_ phase by increasing the copper content in the alloy, effectively above 13% [1]. It is established that the early full strength of the amalgam is achieved within one hour of placement in the prepared tooth cavity [5]. The setting reaction of this alloy is the same as the reaction for the conventional alloy [10]. After the formation of the γ_2_ phase, there is a reaction between this and the silver–copper component, leading to the formation of the copper–tin phase and γ_1_ phase [10,18]. 

The results of the Vickers hardness measurement as shown in Table 2 above for each of the amalgam samples, showed that the hardness of the amalgam increased with the increase in copper content within each of the samples. Such phenomena are, however, independent of time.

### 4.3. Analysis of Energy Released

The energy of formation given off or generated due to the release of mercury vapor can be determined by the following thermodynamic method (Equation (1)):

(1) $$E=\frac{C\;\times \;H}{M}$$

where:

E = Energy of reaction due to vapor released.
C = Recorded concentration of mercury vapor released.
H = Energy given off when one atom of mercury is released (32 kilojoules per mole).
M = Molecular weight of mercury (200.59 g per mole)

The energies given off from each of the amalgams at each stage of measurement are determined from the calculations as indicated in Equation (1). A decrease in energy is observed with time; therefore, it can be concluded that the energy is proportional to the concentration of mercury vapor released from the amalgam, regardless of the brand. Also, the higher the copper content present in the alloy, the less mercury vapor is released from the amalgam.

In accordance with the results generated from the plots, it is determined that the amalgam brands with higher copper percentages tend to release a lower concentration of mercury vapor within a shorter time interval, accounting for the steeper gradient of higher copper levels within the alloy. The energy of formation is indeed lower for the alloy having the higher copper percentage in the alloy composition.

The results of the experimental data, as listed in Table 1, give the values of the concentrations of mercury vapor as given off by various amalgam alloys. From the Dispersalloy (11.8% copper) brand of dental amalgam to the Sybralloy (33% copper), the measurement of mercury vapor shows a decrease, indicating that vapor release is directly proportional to the copper content of the alloy. Additionally, the mercury vapor decreases with an increase in time and attains a somewhat steady-state value. Such steady-state values will be further achieved due to the oxidation of the alloy with the passage of time. 

**Figure 4:**
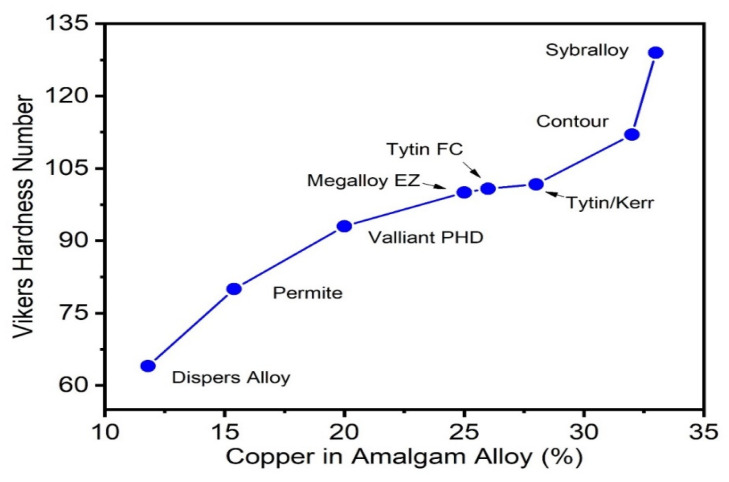
Increase in the Vickers hardness number as a function of copper percentage in the alloy.

Both the Contour (31% Cu) and Sybralloy (33% Cu) brands of amalgam demonstrate the lowest energy of formation, which supports the conclusion that the highest copper content in the alloy produces the least concentration of mercury vapor released from the amalgams. This research has proven the desired results of the experiment. As shown, the hardness of the amalgams increases with increasing copper percentage. The corresponding plot is shown in Figure 4, which shows a graphical representation of the phenomena.

Further investigation regarding the amalgam alloy is required for designing alloys with optimum compositions. In recent years, the American public has become more concerned and ambivalent about the continued mercury vapor release from dental amalgam. Presumably, as more knowledge of this material propagates, there is a high chance that amalgam could become obsolete due to the concerns of the mercury released from such material.

**Figure 5:**
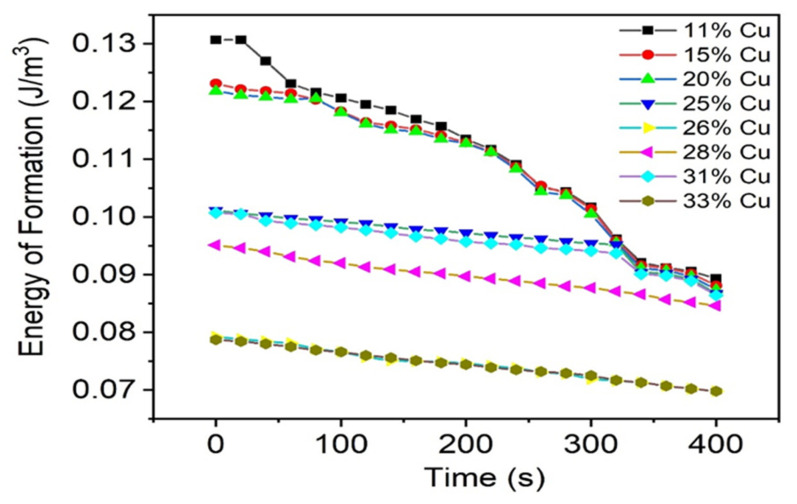
Energy of formation of alloys containing different amounts of Cu as a function of time.

The energy released versus the time plot was tabulated as shown in Figure 5. The energies given off from each of the amalgams were determined from calculations as shown in Equation (1).

## 5. Conclusions

1. A systematic study has been performed to determine the release of mercury vapor from the eight most common brands of dental amalgam. The release of Hg vapor in order of decreasing amount is found to be as follows: Disperesalloy Brand, Permite C/SDI, Valliant PHD, Megalloy EZ, Tytin FC, Tytin/kerr, Contour, and Sybralloy.
2. The hardness of the amalgam is inversely proportional to the mercury vapor released from the alloy (i.e., hardness increases with decreasing copper percentage).
3. X-ray diffraction confirms larger accumulations of γ_2_-phase subsequent to trituration.
4. The amount of energy required for the removal of mercury atoms during vapor release decreases with increasing time duration.
5. A new dental amalgam alloy can be achieved by possibly introducing a new metal to the existing alloy, such as titanium powder.

In brief, this study concludes that the copper content in amalgam is correlated with the amount of Hg vapor released from the alloy. The low-copper amalgam showed higher releases of Hg vapor. In order to manufacture an improved amalgam alloy, either a reduced quantity of mercury or an increased quantity of copper should be considered when designing the alloy composition, along with the introduction of new material if possible. More research and investigations need to be made into the modification of this alloy without significantly affecting its properties.

## Figures and Tables

**Table 1:** Experimental data for mercury vapor concentration (kg/m^3^) released from eight brands of dental amalgam samples.

Time (s)	Disperse Alloy (kg/m^3^)	Permite C/SDI (kg/m^3^)	Valiant PHD (kg/m^3^)	Megalloy EZ (kg/m^3^)	Tytin FC/Kerr (kg/m^3^)	Tytin/Kerr (kg/m^3^)	Contour (kg/m^3^)	Sybralloy (kg/m^3^)
	11.8% Cu	15.4% Cu	20% Cu	25% Cu	26% Cu	28% Cu	31% Cu	(33% Cu)
0	846.085	796.8452	788.7453	654.4776	651.6575	615.6716	513.0673	509.6588
20	838.5543	790.5823	784.0675	651.0781	647.4575	612.7408	510.4512	507.6844
40	821.8564	788.5934	781.8564	648.4752	642.8862	608.8856	507.8546	504.8556
60	796.6544	785.6743	779.5686	645.3237	640.5644	602.5639	505.4364	501.5342
80	787.0785	778.5879	774.9676	643.9465	638.5997	598.4522	499.5089	497.8673
100	780.6533	765.8547	764.6354	641.7498	635.7675	595.4965	496.2395	495.9536
120	773.851	753.6457	750.6793	639.4652	632.7673	591.2894	490.2007	491.8945
140	767.3113	749.4512	744.8476	636.3649	629.5088	588.2098	487.9915	489.6577
160	756.4582	745.463	742.9806	632.8451	625.6554	585.7898	485.9067	485.9676
180	748.9329	738.8486	734.5864	631.9463	622.8789	583.8456	484.773	483.7462
200	734.831	730.6582	729.5655	629.4856	619.7769	580.5487	482.9566	481.6766
220	723.0086	720.7678	719.8867	626.8354	617.3744	578.3008	480.6876	478.5653
240	706.5386	703.5541	700.8723	624.0376	614.8842	575.3065	478.1153	475.7674
260	684.1459	679.7895	675.9665	621.2743	612.3428	573.0678	473.5972	473.8665
280	675.8653	673.8823	671.5434	619.8211	610.8452	569.6458	471.3412	471.8604
300	658.6462	654.6008	650.7685	617.3845	608.4768	567.7342	465.8456	469.6997
320	623.0678	621.0804	618.4437	616.3329	606.5646	563.9647	463.698	464.0767
340	595.9563	591.4318	589.4878	585.5875	583.5453	560.7456	461.9438	461.5682
360	590.5342	589.6074	586.9652	583.7677	581.4436	554.7439	457.8348	458.0634
380	586.6308	583.3116	580.5896	577.3458	575.3876	551.8856	453.1195	454.7683
400	577.8899	570.5078	565.5768	561.6455	559.6763	547.4865	451.7344	451.7554

**Table 2:** Hardness for each amalgam brand with corresponding percent copper in the alloy.

Alloy	Percentage Copper	Hardness
Disperse Alloy	11.8	64HV200
Permite	15.4	80HV200
Valliant PHD	20	93HV200
Megalloy EZ	25	100HV200
Tytin FC	26	100.8HV200
Tytin/Kerr	28	101.7HV200
Contour	31	112HV200
Sybralloy	33	129HV200

## Data Availability

The data that support the findings of this study are available from the corresponding author upon reasonable request.
